# Tubeimoside-1: A review of its antitumor effects, pharmacokinetics, toxicity, and targeting preparations

**DOI:** 10.3389/fphar.2022.941270

**Published:** 2022-07-15

**Authors:** Chang-Lin Wang, Ming-Zhou Gao, Dong-Mei Gao, Ying-Hui Guo, Zhan Gao, Xiang-Ju Gao, Jie-Qiong Wang, Ming-Qi Qiao

**Affiliations:** ^1^ School of Chinese Medicine, Shandong University of Traditional Chinese Medicine, Jinan, China; ^2^ Research and Innovation Team of Emotional Diseases and Syndromes in Shandong University of Traditional Chinese Medicine, Jinan, China; ^3^ Institute of Traditional Chinese Medicine Innovation, Shandong University of Traditional Chinese Medicine, Jinan, China; ^4^ School of Pharmacy, Shandong University of Traditional Chinese Medicine, Jinan, China

**Keywords:** tubeimoside-1, antitumor, pharmacokinetics, toxicity, targeting preparations

## Abstract

Tubeimoside-1 (TBMS-1), a natural triterpenoid saponin found in traditional Chinese herbal medicine Bolbostemmatis Rhizoma, is present in numerous Chinese medicine preparations. This review aims to comprehensively describe the pharmacology, pharmacokinetics, toxicity and targeting preparations of TBMS-1, as well the therapeutic potential for cancer treatement. Information concerning TBMS-1 was systematically collected from the authoritative internet database of PubMed, Web of Science, and China National Knowledge Infrastructure applying a combination of keywords involving “tumor,” “pharmacokinetics,” “toxicology,” and targeting preparations. New evidence shows that TBMS-1 possesses a remarkable inhibitory effect on the tumors of the respiratory system, digestive system, nervous system, genital system as well as other systems *in vivo* and *in vitro*. Pharmacokinetic studies reveal that TBMS-1 is extensively distributed in various tissues and prone to degradation by the gastrointestinal tract after oral administration, causing a decrease in bioavailability. Meanwhile, several lines of evidence have shown that TBMS-1 may cause adverse and toxic effects at high doses. The development of liver-targeting and lung-targeting preparations can reduce the toxic effect of TBMS-1 and increase its efficacy. In summary, TBMS-1 can effectively control tumor treatment. However, additional research is necessary to investigate *in vivo* antitumor effects and the pharmacokinetics of TBMS-1. In addition, to reduce the toxicity of TBMS-1, future research should aim to modify its structure, formulate targeting preparations or combinations with other drugs.

## 1 Introduction

**FIGURE 1:**
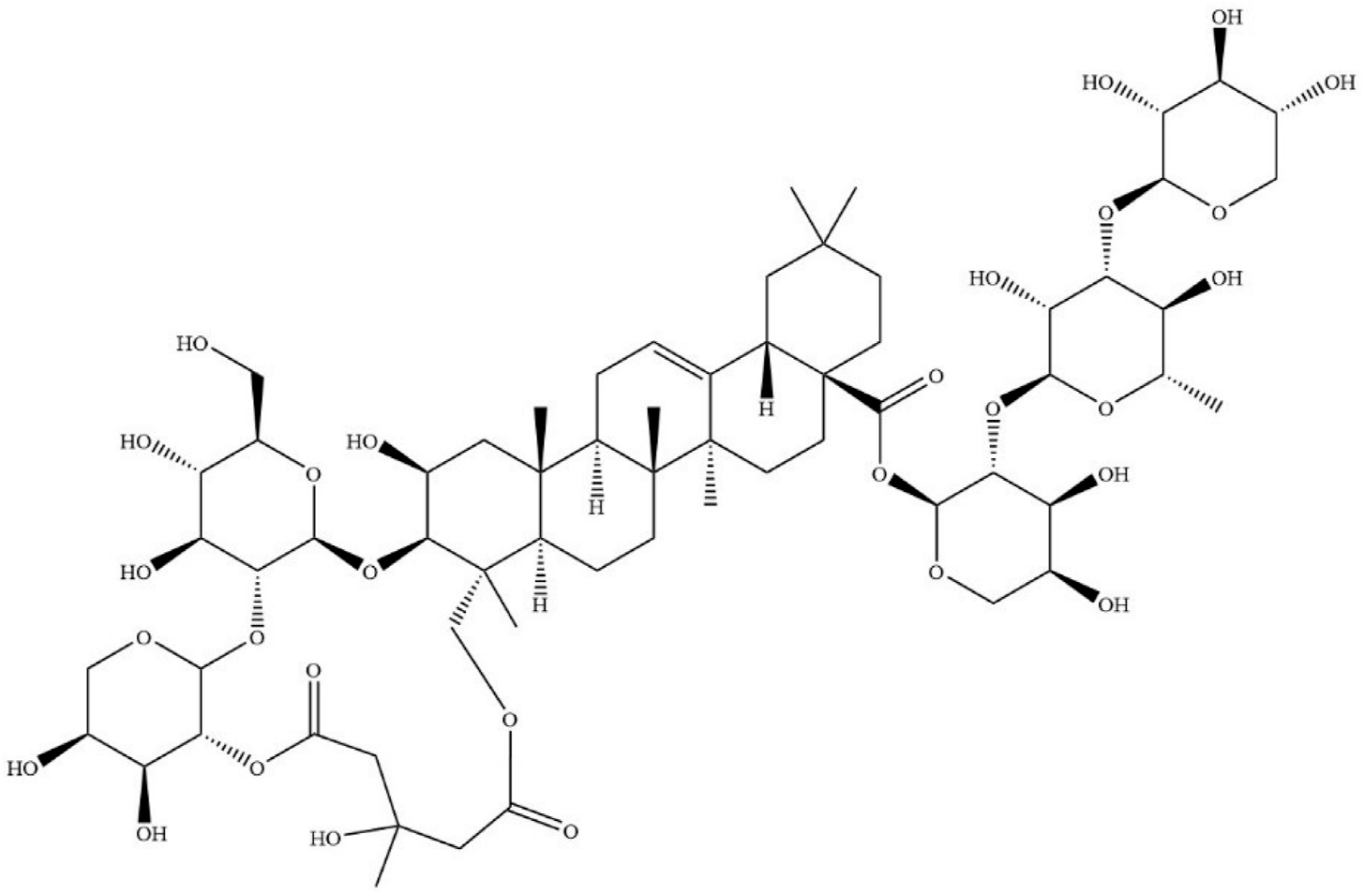
Chemical structure of TBMS-1.

Dried tubers of *Bolbostemma paniculatum* (Maxim.) Franquet, a member of *Fritillaria* genus and *Cucurbitaceae* family are drug sources of Bolbostemmatis Rhizoma. Bolbostemmatis Rhizoma was first recorded in *Ben-Cao-Zheng-Yao*. It has detoxification effects, dispersing knots as well as detumescence, and is primarily used for treatment of diseases, including mastitis, scrofula and phlegmatic nodule among others (Zhu et al., 2018). Tubeimoside-1 (TBMS-1, CAS No.102040-03-9) (Figure 1) is a triterpenoid saponin extracted from Bolbostemmatis Rhizoma (Li et al., 2021). Its contents in medicinal materials and proprietary Chinese medicines are shown in Table 1.

**TABLE 1:** The content of TBMS-1 in herbal medicine and proprietary Chinese medicine.

Name	Content/Concentration	References
Bolbostemmatis Rhizoma	14.96 mg/g	Huang et al. (2016)
Ruzengning capsules	8.97 mg/g	Sheng et al. (2018)
Jiazhongxiao preparation	995.30 μg/g	Cui et al. (2008)
Jibei Xiaozhong capsules	6.90 mg/g	Lu et al. (2015)
Shuangbei capsules	0.33 mg/g	Quan et al. (2018)

Malignant tumors are associated with high morbidity and mortality. This necessitates the development of safe and effective treatments for cancer management. Natural compounds, which are characterized by a high efficacy and limited side effects, are potential chemoprophylaxis and chemotherapeutic options (Kong et al., 2021). TBMS-1 exerts anticancer effects in multiple tumors *via* targeting multiple targets and pathways (Wang et al., 2021b). Despite its antitumor activities, its toxic effects have yet to be fully elucidated. Reports indicate that TBMS-1 is toxic to the liver and spleen. Targeting preparations are aimed at maintaining effective concentrations, reducing the times of drug use and side effects. Preparation of TBMS-1 into a targeting preparation mediate synergism and detoxification. Drug pharmacokinetics are closely correlated with pharmacology, toxicology, and targeting preparations of drugs. Drug efficacy, safety and rational use are informed by in-depth analysis of their absorption, distribution, metabolism, and excretion (Liu, 2016). This review summarizes the recent research progress in antitumor effects, toxicity, pharmacokinetics, and targeting preparations of TBMS-1. Our findings elucidate on its therapeutic potential and provide a reference for the development of novel, safe and effective anticancer drugs.

## 2 Review methodology

A systematic search of literature was performed according to the preferred reporting items for systematic reviews and meta-analyses (PRISMA) guidelines. The search for literature was obtained from electronic databases, including the China National Knowledge Infrastructure (CNKI), PubMed, Web of Science, and Google Scholar. The keywords used for the search were: “tubeimoside-1,” “tubeimoside Ⅰ,” “tubeimoside A,” “tumor,” “pharmacokinetics,” “toxicity,” and “targeting preparations.” Additional information in PhD and MSc publications in China were also collected from the CNKI database. Information on antitumor effects, pharmacokinetics, toxicity and targeting preparations of TBMS-1 in the obtained information was obtained.

The inclusion criteria were studies: 1) Published in either English or Chinese; 2) On antitumor effects of TBMS-1; 3) On pharmacokinetics of TBMS-1; 4) Reporting on toxicity of TBMS-1. 5) Reporting on targeting preparations of TBMS-1. The exclusion criteria were: 6) Conference abstracts, letters to editors, case reports, systematic reviews, and meta-analysis studies. 106 papers reporting on antitumor effects, pharmacokinetics, toxicity, and targeting preparations of TBMS-1 were reviewed.

## 3 Antitumor effects

**FIGURE 2:**
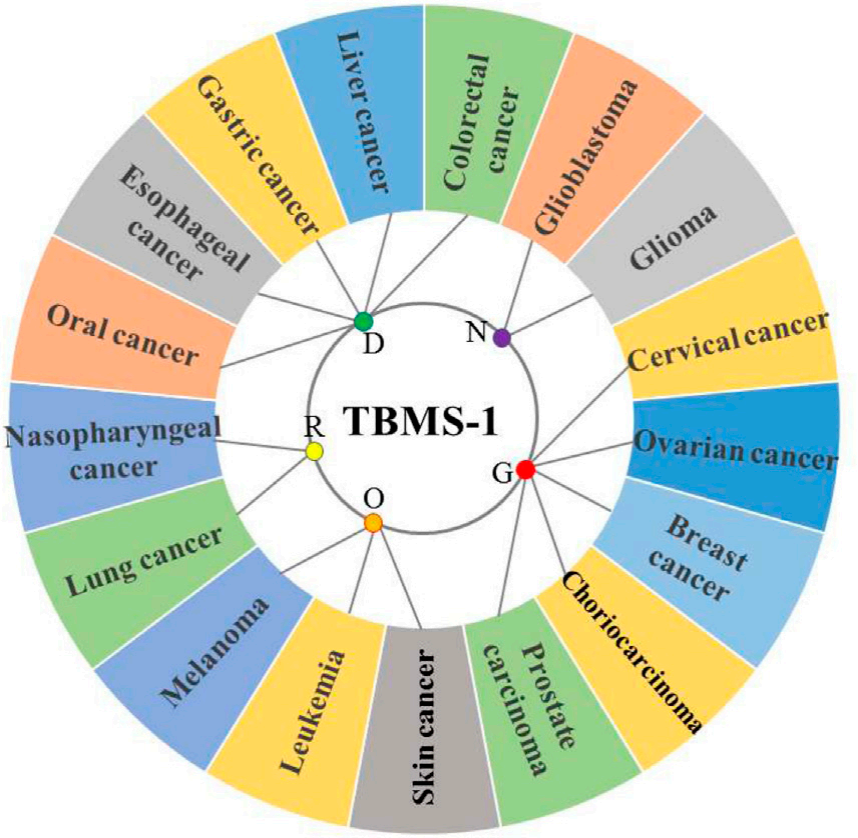
Antitumor activity of TBMS-1 on different physiological systems (R = respiratory system tumor, D = digestive system tumor, N = nervous system tumor, G = genital system tumor, O = other system tumors).

TBMS-1 has cytotoxic effects on different tumor cells. Most of the studies have been primarily performed using cellular models *in vitro*, with limited *in vivo* studies (Figure 2; Tables 2, 3 **)**.

**TABLE 2:** Antitumor activity of TBMS-1.

Tumor type	Cell lines/Model	Activity/mechanisms of action	Application	References
Lung cancer	NCI-H1299 cells	Overexpression of miR-126-5p inactivated the VEGF-A/VEGFR2/ERK signaling pathway to inhibit the activity of NCI-H1299 cells	*In vitro*	(Shi et al., 2018a; Shi et al., 2018b)
	NCI-H1299 cells and NCI-H1975 cells	Disrupted the interaction between mitochondrial and lysosomal pathways leading to the killing of lung cancer cells	*In vitro/in vivo*	Wang et al. (2020a)
	A549 cells	Inhibited the proliferation and induced apoptosis of cells by increasing the Bax to Bcl-2 ratio, and decreasing COX-2 expression	*In vitro*	Zhang et al. (2011)
	A549 and PC9 cells	Decreased cell proliferation and expression of cell growth-associated proteins, such as p21, p15, and Cyc B1, upregulated expression of proapoptotic factors, Bax and cleavage of procaspase-3, downregulated expression of antiapoptotic factors, Mcl-1 and cIAP-1, and activated the MAPK-JNK signaling pathway	*In vitro*	Hao et al. (2015)
	NCL-H460 cells and NCL-H460 xenograft mice model	Stimulated the proteasomal degradation of VEGFR2 and Tie2, leading to inhibition of AKT/mTOR signaling	*In vitro/in vivo*	Gu et al. (2016)
	NCL-H460 cells	Exerted cytotoxicity in NCI-H460 lung cancer cells through nucleolar stress-induced p53/MDM2, mTOR, and NF-κB signaling pathways	*In vitro*	Lin et al. (2016)
	A549 cells	Increased the activity of Caspase-3, Caspase-9, and Parp to induce apoptosis	*In vitro*	Su et al. (2021)
	PGCL3 cells	Reduced the secretion and activity of MMP-2, and decreased the adhesion rate of laminin and fibronectin to influence the invasion and adhesion of PGCL3 cells	*In vitro*	Yu et al. (2008)
	Lewis lung cancer spontaneous metastasis mice model	Downregulated the expression of metastasis-promoting genes *CD44v6* and *ErBb-2*, and upregulated the expression of metastasis-suppressing gene, nm23-H1	*In vivo*	Wang et al. (2006a)
	A549/DDP cells	Upregulated the expression of p53 and Caspase-3, and reduced the ratio of Bcl-2/Bax to enhance the sensitivity of cancer cells to cisplatin	*In vitro*	Wang et al. (2020b)
Nasopharyngeal cancer	CNE-2Z cells	Inactivated Bcl-2, activated Bax and MAPK to induce tumor cell apoptosis	*In vitro*	(Weng et al., 2003; Liu et al., 2007)
Oral cancer	SCC15 cells and CAL27 cells	Induced intrinsic apoptosis through the ERK1/2/Bcl-2/Caspase-9/Caspase-3/PARP pathway, and the extrinsic apoptotic by decreasing the expression of Caspase-3, Caspase-7, and Caspase-8, and inhibited metastasis by decreasing c-Myc and MMP-7 protein expression	*In vitro*	Wu et al. (2018)
Esophageal cancer	EC109 cells	Mitochondria-induced intrinsic apoptosis and p21/Cyc B1/cdc2 complex-related G2/M cell cycle arrest	*In vitro*	Xu et al. (2013)
Gastric cancer	BGC823 cells	Upregulated the expression of Bax and downregulated expression of Bcl-2 to increase the ratio of Bax to Bcl-2	*In vitro*	Zhang et al. (2013)
Liver cancer	HepG2 cells	Promoted apoptosis signaling pathways and production of reactive oxygen species by regulating TNF-α, NF-κB, JNK, and p53	*In vitro*	(Wang et al., 2011b; Yin et al., 2011)
	HepG2 cells	Activated AMPK signaling pathway, increased the expression of Beclin 1, and LC3-Ⅱ/LC3-Ⅰ to induce autophagy	*In vitro*	Ruan et al. (2020)
	HepG2 cells	Reduced the expression and activity of MMP-2 and MMP-9 proteins in HepG2 cells to inhibit cell migration and invasion	*In vitro*	Zhong et al. (2016)
Colorectal cancer	SW480 and HCT116 cells	Initiated autophagy by activating ROS/AMPK signaling and impaired autophagy flux by = inhibiting lysosomal proteolysis activity	*In vitro*	Yan et al. (2019)
	SW480 and HCT-8 cells	Inhibited cell proliferation and invasion via suppressing the Wnt/β-catenin signaling pathway	*In vitro*	Bian et al. (2015)
Glioblastoma	U87, LN229 cells and U87 xenograft mice model	Decreased the protein level of MET by increasing its ubiquitination degradation to inhibit proliferation, migration, and invasion of glioblastoma cells	*In vitro/in vivo*	Cao et al. (2019)
Glioma	U251 cells	Increased Bax/Bcl-2 and the concentration of ROS by promoting the release of cytochrome C and activation of Caspase-3	*In vitro*	Jia et al. (2015)
	U251 cells	Downregulated the expression of miR-21 and upregulated that of PDCD4 to induce cell apoptosis	*In vitro*	Jia et al. (2016)
	U251 cells	Upregulated the expression of FADD, Caspase-8, and Caspase-3 proteins to induce apoptosis	*In vitro*	Jia et al. (2014)
	U251 cells	Inhibited DNA synthesis and induced G2/M phase arrest by targeting the PI3K/Akt/p21 and the CDK1/Cyc B1 signaling cascades	*In vitro*	Cao et al. (2020)
Cervical cancer	HeLa cells	Decreased mitochondrial membrane potential, increased Cytc and Bax/Bcl-2	*In vitro*	(Ma et al., 2004; Wang et al., 2005; Wang et al., 2006b)
	HeLa cells	Induced the depletion of mitochondrial transmembrane potential and activated the Caspase pathway to cause cell death. It activated the UPR signaling pathway to induce cytotoxic effects	*In vitro*	Xu et al. (2009)
	HeLa cells	Activated AMPK to initiate autophagy, blocked autophagic flux, and increased levels of damaged autophagy lysozyme to aggravate cytotoxic activity	*In vitro*	Feng et al. (2018)
Ovarian cancer	A2780/DDP cells	Downregulated ERK1/2 and upregulated p38 signaling pathway to increase the inhibitory effect of cisplatin on A2780/DDP cells proliferation	*In vitro*	(Liu et al., 2011a; Liu et al., 2011b)
	SKOV-3 cells	Increased Bax expression by inducing phosphorylation of p38 and MAPK and decreased Bcl-2 levels by reducing the phosphorylating of ERK1/2 to activate Caspase-3	*In vitro*	Chen et al. (2012)
Breast cancer	MDA-MB-231 cells and MDA-MB-231 xenograft mice model	Inhibited the binding ability of NF-κB to the promoter of CXCR4 to reduce the expression of CXCR4 and metastasis of breast cancer cells	*In vitro/in vivo*	Peng et al. (2016)
	MDA-MB-231 cells	Induced autophagy through the PI3k-Akt-mTOR signal pathway	*In vitro*	(Jiang et al., 2019; Liu et al., 2019)
Choriocarcinoma	JEG-3 cells	Induced mitochondrial dysfunction and regulated the p38/MAPK, ERK1/2 and PI3K/Akt signaling pathways	*In vitro*	Huang et al. (2011)
Prostate carcinoma	DU145 cells	Induced mitochondrial apoptosis by increasing ROS generation, mitochondrial dysfunction, endoplasmic reticulum stress, Bcl-2 family protein and cleaved caspase-3 expression, and activating ASK-1 and its downstream targets p38 and JNK.	*In vitro*	Yang et al. (2016)
Skin cancer	SCL-1 cells	Downregulated circ_0000376 to promote the expression of miR-203 to inhibit the proliferation, migration, and invasion of skin cancer cells, and accelerate cell apoptosis	*In vitro*	Wang et al. (2021a)
Leukemia	HL-60 cells	Decreased the expression of Cyc B1 and blocked the cell cycle	*In vitro*	Hu et al. (2003)
	HL-60 cells	Induced the differentiation of HL-60 cells to more mature cells with the functional characteristics of granulocytes	*In vitro*	Yu et al. (1996)
Melanoma	A375 cells	Blocked the cell cycle in G2/M phase and induced apoptosis of A375 cells by downregulating Bcl-2 and upregulating Bax	*In vitro*	Rasul et al. (2012)
	MV3, A375 cells and MV3 xenograft mice model	Inhibited cell proliferation through rapid hyperactivation of MEK1/2-ERK1/2 cascade by promoting PTP1B	*In vitro/in vivo*	Du et al. (2020)
	A375 cells and B16 melanoma cells xenograft mice model	Bound to mTOR kinase to suppress activation of mTORC1 leading to disruption of PD-1/PD-L1 interaction and enhanced cytotoxic killing of cancer cells by T cells by decreasing the abundance of PD-L1	*In vitro/in vivo*	Liu et al. (2021)

**TABLE 3:** The IC_50_ value of TBMS-1 in various types of tumor cells.

Cell line	IC_50_ value	Application	References
NCI-H1299 lung cancer cells	17.53 μmol/L	*In vitro*	Wang et al. (2020a)
NCI-H1975 lung cancer cells	25.01 μmol/L	*In vitro*	Wang et al. (2020a)
A549 lung cancer cells	12.30 μmol/L	*In vitro*	Hao et al. (2015)
PC9 lung cancer cells	10.20 μmol/L	*In vitro*	Hao et al. (2015)
NCI-H460 lung cancer cells	23.30 μmol/L	*In vitro*	Gu et al. (2016)
PGCL_3_ lung cancer cells	15.70 μmol/L	*In vitro*	Yu et al. (2008)
A549/DDP cells	12.44 μg/ml	*In vitro*	Wang et al. (2020b)
CNE-2Z nasopharyngeal cancer cells	32.5 μmol/L	*In vitro*	Weng et al. (2003)
SCC15 oral cancer cells	11.6 μmol/L	*In vitro*	Wu et al. (2018)
CAL27 oral cancer cells	14.6 μmol/L	*In vitro*	Wu et al. (2018)
EC109 esophageal cancer cells	45 μmol/L	*In vitro*	Xu et al. (2013)
HepG2 liver cancer cells	15.5 μmol/L	*In vitro*	Wang et al. (2011b)
U251 glioma cells	31.55 μg/ml	*In vitro*	Cao et al. (2020)
HeLa cervical cancer cells	34.8 μmol/L	*In vitro*	Wang et al. (2006b)
A2780/DDP cells	16.10 μmol/L	*In vitro*	Liu et al. (2011b)
SKOV-3 ovarian cancer cells	16 μmol/L	*In vitro*	Chen et al. (2012)
Choriocarcinoma JEG-3 cells	8.5 μmol/L	*In vitro*	Huang et al. (2011)
Prostate carcinoma DU145 cells	10 μmol/L	*In vitro*	Yang et al. (2016)
Prostate carcinoma PC3 cells	20 μmol/L	*In vitro*	Yang et al. (2016)
Leukemia K562 cells	19.7 μmol/L	*In vitro*	Liu et al. (2006)
Leukemia HL-60 cells	2.4 μmol/L	*In vitro*	Yu et al. (1996)
Melanoma MV3 cells	12 μmol/L	*In vitro*	Du et al. (2020)
Melanoma A375 cells	8 μmol/L	*In vitro*	Du et al. (2020)

### 3.1 Respiratory system tumors

#### 3.1.1 Lung cancer

Lung cancer is a prevalent malignant tumor whose incidence rates are directly correlated with smoking. TBMS-1 has specific killing effects on lung cancer cells. It exerts dual anticancer effects by disrupting mitochondrial and lysosomal pathways as well as their interactions (Wang et al., 2020a). *In vitro*, exactly 10 μmol/L TBMS-1 can inactivate vascular endothelial growth factor-A/vascular endothelial growth factor receptor 2/extracellular signal-regulated kinase (VEGF-A/VEGFR2/ERK) signal pathway by overexpressing miR-126-5p, effectively inhibiting NCI-H1299 cell proliferation and metastasis as well as promoting their apoptosis (Shi et al., 2018a; Shi et al., 2018b). Additionally, TBMS-1 (4, 8, 12 μmol/L) induced the apoptosis of A549 cells by downregulating B-cell lymphoma-2/BCL2-Associated X (Bcl-2/Bax) and suppressing cyclooxygenase-2 (COX-2) expressions (Zhang et al., 2011). 1 μg/ml TBMS-1 significantly increased the activities of cysteinyl aspartate specific proteinase 9 (Caspase-9), Caspase-3, and poly ADP-ribose polymerase (PARP) in A549 cells, implying that TBMS-1 induces apoptosis via the Caspase signaling pathway (Su et al., 2021). Moreover, TBMS-1 (8, 16 μmol/L) downregulated p21, p15 and cyclin B1(Cyc B1) levels in A549 and PC9 cells; blocked the cells in the G2/M phase; activated mitogen-activated protein kinase/c-Jun N-terminal kinase (MAPK/JNK) signaling pathways to inhibit cell proliferation; upregulated the cleavage of Bax and procaspase-3; downregulated the expressions of myeloidcellleukemia-1 (Mcl-1) and inhibitors of apoptotic protein (cIAP-1), thereby inducing cell apoptosis (Hao et al., 2015). TBMS-1 (1.25–5 μmol/L) inhibited the adhesion, invasion, and migration of human highly metastatic giant cell lung cancer PGCL3 cells, which decreases cell adhesion to laminin and fibronectin and the secretion and Matrix metalloproteinase-2 (MMP-2) activity (Yu et al., 2008). Researchers identified seven differentially expressed proteins in NCI-H460 cells treated with 20 μmol/L TBMS-1 for 9 h. Functionally, TBMS-1 cytotoxicity involved nucleolar stress-induced p53/murinedoubleminuteclone2 (MDM2), mammalian target of rapamycin (mTOR) and nuclear factor-κ-gene binding (NF-κB) signaling pathways (Lin et al., 2016). Cisplatin is a traditional anticancer drug that easily triggers drug resistance when used for prolonged periods of time. Cisplatin (10 μg/ml) combined with TBMS-1 (10 μg/ml) were associated with a high sensitivity of A549/DDP cells (cisplatin-resistant non-small cell lung cancer cells) to cisplatin (Wang et al., 2020b).

*In vivo*, researchers subcutaneously inoculated NCI-H1299 cells into nude mice and intraperitoneally injected them with 4 mg/kg TBMS-1. Tumor volumes and weights in the treatment group were significantly reduced (Wang et al., 2020a). In another study, microvessel densities around and in the center of non-small cell lung cancer (NSCLC) xenograft mice models treated with 5 mg/kg TBMS-1 were significantly low than in the blank control group, confirming that TBMS-1 effectively inhibits tumor growth and vascularization *in vivo* (Gu et al., 2016; Li et al., 2016). TBMS-1 significantly inhibited Lewis lung cancer proliferation and spontaneous metastasis in nude mice by downregulating metastasis-promoting genes (*CD44v6* and *ErBb-2*) and upregulating the metastasis suppressor gene, nm23-H1 (Wang et al., 2006a).

#### 3.1.2 Nasopharyngeal cancer

TBMS-1 (50 μmol/L) resulted in typical programmed cell death in morphology and biochemistry in human nasopharyngeal cancer CNE-2Z cells *in vitro*. TBMS-1-induced CNE-2Z cell apoptosis was attributed to Bcl-2 inactivation and Bax activation (Weng et al., 2003). Elsewhere, MAPK activities in CNE-2Z cells treated with TBMS-1 were evaluated by autoradiography, liquid scintillation, and Western blotting. It was established that TBMS-1 could rapidly activate MAPK, thereby inducing tumor cell apoptosis (Liu et al., 2007).

### 3.2 Digestive system tumors

#### 3.2.1 Oral cancer

Globally, oral cancer is the 6th most prevalent cancer, and oral squamous cell carcinoma is the most common subtype (Radhika et al., 2016). Using two oral squamous cancer cell lines (CAL27 and SCC15), it was reported that TBMS-1 significantly dose- and time-dependently suppressed the proliferation of cells. Moreover, a high concentration of TBMS-1 (10 and 15 μmol/L) inhibited cell migration by downregulating the cellular-myelocytomatosis viral oncogene (c-Myc) and MMP-7 expressions. Besides, TBMS-1 stimulates the ERK1/2/Bcl-2/Caspase-9/Caspase-3/PARP signaling pathway to induce endogenous apoptosis, and downregulates Caspase-3, Caspase-7, and Caspase-8 expression to induce exogenous apoptosis (Wu et al., 2018).

#### 3.2.2 Esophageal cancer

Esophageal cancer incidences are significantly high in developing countries, specifically in China. TBMS-1 (30–45 μmol/L) inhibited the proliferation and induced the apoptosis of esophageal cancer EC109 cells *in vitro*. Altered proteins in the nucleus of EC109 cells correlated with mitochondrial function and cell proliferation. Furthermore, TBMS-1-induced molecular events were associated with mitochondria-induced intrinsic apoptosis and p21/cyclin B1/cell division cycle 2 (p21/Cyc B1/cdc2) complex-related G2/M cell cycle arrest (Xu et al., 2013).

#### 3.2.3 Gastric cancer

Globally, gastric cancer is the 4th most prevalent tumor type and the 2nd most common cause of cancer-related mortalities (Compare et al., 2010). Researchers found that TBMS-1 (5–20 μmol/L) inhibited the proliferation of gastric cancer BGC823 cells in a concentration- and time-dependent manner, and induced their apoptosis by regulating the Bcl-2 gene family (Zhang et al., 2013).

#### 3.2.4 Liver cancer

In the past 10 years, liver cancer has been the 5th most common malignant tumor in men and the 8th most prevalent malignant tumor in women (Bosch et al., 2004). *In vitro*, TBMS-1 (10–30 μmol/L) mediated various apoptotic signaling pathways, including activating Caspase3 and 9, as well as increasing the Bax/Bcl-2 ratio among others, thereby effectively inhibiting hepatoma HepG2 cell proliferations (Wang et al., 2011b). Additionally, TBMS-1 induced the production of reactive oxygen species *via* signal regulation of tumor necrosis factor *a* (TNF-α), NF-κB, JNK as well as p53 and blocking the cell cycle at the G2/M phase, thereby promoting cell apoptosis (Yin et al., 2011). Microscopic imaging and fluorescence spectroscopic analysis of apoptosis in HepG2 cells before and after TBMS-1 treatment showed that TBMS-1 treatment promoted HepG2 cell apoptosis and inhibited cells proliferation, particularly in the G2/M phase (Lin et al., 2014). TBMS-1 (8–16 μmol/L) dose-dependently enhanced AMP-activated protein kinase (AMPK) phosphorylation, thereby activating the AMPK signaling pathway and inducing autophagy in HepG2 cells, as evidenced by autophagosome accumulation and upregulated expressions of Beclin1 and LC3-Ⅱ/LC3-Ⅰ (Ruan et al., 2020). Besides, TBMS-1 (15–30 μmol/L) significantly downregulated the expressions as well as activities of MMP-2 and MMP-9 proteins in HepG2 cells, thereby inhibiting their migration and invasive abilities (Zhong et al., 2016).

#### 3.2.5 Colorectal cancer

TBMS-1 inhibits colorectal cancer cell proliferation and enhances their apoptosis. TBMS-1 activates reactive oxygen species/AMP-activated protein kinase (ROS/AMPK) signaling to promote autophagic initiation, and blocks the autophagy flux by inhibiting lysosomal hydrolytic enzymes, resulting in massive impaired autophagy-lysosome accumulation to trigger cell apoptosis. Low TBMS-1 doses combined with 5-fluorouracil (5-FU) and Doxorubicin (DOX) exhibited better cytotoxic effects, compared to single treatments (Yan et al., 2019). In another study, TBMS-1 (10–50 μg/ml) stimulated the Wnt/β-catenin signaling pathway to suppress colorectal cancer cell proliferation and invasion (Bian et al., 2015).

### 3.3 Nervous system tumors

#### 3.3.1 Glioblastoma

Glioblastoma, the most common subtype of malignant glioma, has high metastasis and recurrence rates with poor prognosis after radiotherapy and chemotherapy (Ostrom et al., 2021). Due to gene amplifications, mesenchymal to epithelial transition factor (MET) is abnormally activated in glioblastoma, resulting in activation of the downstream pathway and a significant increase in cell proliferations, migration, and invasion. TBMS-1 enhances ubiquitin degradation of MET and regulates the expressions of key proteins in pathways related to proliferation, migration and invasion of glioblastoma cells. Thus, MET is an important target for TBMS-1, and TBMS-1 has the potential for being a targeting monomer of traditional Chinese medicine (Cao et al., 2019).

#### 3.3.2 Glioma

Glioma is a prevalent invasive malignant tumor of the central nervous system, with high recurrence and mortality rates as well as low cure rates. Despite advances in diagnosis and treatment of gliomas, the overall survival rate is significantly low. Thus, development of novel drugs is essential to avert this situation. TBMS-1 is a potential effective treatment option for gliomas. *In vitro*, 15–25 μg/ml TBMS-1 upregulated Bax/Bcl-2 and ROS levels by releasing cytochrome C (Cytc) and activating Caspase-3, resulting in glioma U251 cell apoptosis (Jia et al., 2015). In another study, TBMS-1 downregulated miR-21 expressions and upregulated the protein expressions of its target gene (*PDCD4*) to induce U251 cell apoptosis (Jia et al., 2016). Moreover, 10–25 μg/ml TBMS-1 exerted its toxic effects on U251 cells by upregulating Fas-associated death domain (FADD), Caspase-8, and Caspase-3 protein levels (Jia et al., 2014). Cao et al. showed that TBMS-1 (20–40 μg/ml) suppressed the survival rate of U251 cells by inhibiting protein kinase B (AKT) phosphorylation. Moreover, TBMS-1 inhibited DNA synthesis and induced G2/M phase arrest by targeting phosphatidylinositol 3 kinase/protein kinase B/p21 (PI3K/AKT/p21) and cyclin-dependent kinase1/cyclin B1 (CDK1/Cyc B1) signaling pathways. Thus, TBMS-1 inhibits glioma progression by inhibiting the PI3K/AKT pathway (Cao et al., 2020).

### 3.4 Genital system tumors

#### 3.4.1 Cervical cancer

Cervical cancer is a prevalent and deadly malignant tumor among women. TBMS-1 inhibits cervical cancer cell proliferation and exhibits synergistic effects with cisplatin, which reduces cisplatin dose (Zhang et al., 2010). A previous study used a transmission electron microscope to examine 10–50 μmol/L TBMS-1-induced ultrastructural changes in cervical cancer HeLa cells. They found that HeLa cells exhibited a series of apoptotic changes, including mitochondrial swelling and degeneration, which explains the occurrence of the endogenous apoptotic pathway centered on mitochondria from the morphologic perspective (Wang et al., 2007). The apoptosis of cervical cancer HeLa cells induced by TBMS-1 decreases the mitochondrial membrane potential and upregulates Cytc released into the cytoplasm, and that of Bax/Bcl-2 (Ma et al., 2004; Wang et al., 2005; Wang et al., 2006b).

Proteomic techniques have been used to detect cytotoxic effects of TBMS-1 on HeLa cells. HeLa cells treated with TBMS-1 exhibited significant changes in proteins associated with energy metabolism, protein synthesis, and folding, suggesting that the mitochondria and the endoplasmic reticulum mediate TBMS-1-induced apoptosis. In addition, TBMS-1 depletes mitochondrial transmembrane potential and activates the Caspase pathway resulting in cell death. TBMS-1 can also activate the unfolded protein response (UPR) signaling pathway and exert cytotoxic effects via activating the endoplasmic reticulum stress pathway and enhancing C/EBP-homologous protein levels or growth arrest and DNA damage-inducible *gene153* (CHOP/GADD153) expression (Xu et al., 2009). Subcellular proteomic analysis in the cytoplasm and membrane protein fractions extracted from HeLa cells revealed that proteins that act as mediators of ROS generation and Ca^2+^ regulation were substantially disrupted in expressions upon TBMS-1 exposures (Xu et al., 2011).

As a novel lethal impaired autophagolysosome inducer, TBMS-1 improves the therapeutic effects of chemotherapeutic drugs, including cisplatin and paclitaxel against cervical cancer. TBMS-1 initiates autophagy by activating AMPK, impairing lysosomal cathepsin activities and blocking the autophagic flux that results in accumulation of impaired autophagolysosomes, aggravating the cytotoxic activities of TBMS-1 on cervical cancer cells (Feng et al., 2018).

#### 3.4.2 Ovarian cancer

Ovarian cancer is the most common, fatal gynecological malignant tumor. Currently, it is treated using various antitumor drugs, including cisplatin, which are associated with high resistance levels. Thus, there is a need to develop novel chemotherapeutic drugs with higher activities and response rates for ovarian cancer treatment. TBMS-1 (8 μmol/L) significantly inhibited A2780/DDP cell proliferations (cisplatin-resistant ovarian cancer cells) by downregulating the ERK1/2 and upregulating the p38 signaling pathway in combination with 6 μmol/L cisplatin (Liu et al., 2011a; Liu et al., 2011b). In another study, TBMS-1-induced ovarian cancer SKOV-3 cell apoptosis was associated with endoplasmic reticulum stress. By activating p38/MAPK, Bax expressions were upregulated and phosphorylated ERK1/2 downregulated Bcl-2 expressions, thereby altering the Bax/Bcl-2 protein ratios and activating the downstream Caspase-3 signal, resulting in apoptosis (Chen et al., 2012).

#### 3.4.3 Breast cancer

Globally, breast cancer is the leading cause of cancer-related death among women. Over 90% of breast cancer-associated deaths are due to metastasis (Geiger and Peeper, 2009). The stromal cell-derived factor-1 (CXCL12) activates CXC chemokine receptor-4 (CXCR4) and promotes tumor cell migration as well as invasion (Zeelenberg et al., 2003). CXCR4 downregulation or blocking of the CXCR4/CXCL12 signaling pathway inhibits tumor metastasis (Richert et al., 2009; Yadav et al., 2010). Peng et al. (2016) confirmed that TBMS-1 inhibits the binding capacity of NF- κB to the CXCR4 promoter *in vivo* and *in vitro*, thereby suppressing CXCR4 expressions in breast cancer cells and inhibiting their metastasis. Moreover, TBMS-1 (4–8 μmol/L) inhibited breast cancer MDA-MB-231 cell proliferation and induced their apoptosis as well as autophagy. Autophagic induction of TBMS-1 might be achieved by regulating the PI3k/Akt/mTOR signaling pathway (Jiang et al., 2019; Liu et al., 2019).

#### 3.4.4 Choriocarcinoma

Choriocarcinoma is a prevalent malignant tumor of the reproductive system, with chemotherapy as its primary clinical treatment. The current anticancer chemotherapeutic methods clear cancer cells *via* inducing their apoptosis, which involves the role of mitochondria in the apoptotic endogenous pathway. TBMS-1 effectively induced apoptosis in choriocarcinoma JEG-3 cells by initiating mitochondrial dysfunction and regulating p38/MAPK, ERK1/2, as well as PI3K/Akt signaling pathways (Huang et al., 2011).

#### 3.4.5 Prostate carcinoma

Prostate carcinoma is a prevalent malignant tumor of the male genitourinary system. Surgery, postoperative radiotherapy, and endocrine therapy are common therapeutic approaches for prostate tumors. Despite various treatments, survival and prognostic outcomes for prostate cancer patients are not satisfactory. *In vitro*, TBMS-1 (5–50 μmol/L) markedly dose-dependently suppressed prostate cancer PC3 cell proliferations. The morphology of prostate cancer cells treated with TBMS-1 exhibited typical apoptotic morphological changes (Wang et al., 2016). In prostate cancer DU145 cells, TBMS-1 (5–100 μmol/L) induced mitochondrial apoptosis as evidenced by ROS generation, mitochondrial dysfunction, endoplasmic reticulum stress, modulated Bcl-2 family proteins and cleaved Caspase-3, as well as activated apoptotic signal-regulated kinase 1 (ASK-1) and its downstream targets (p38 and JNK) (Yang et al., 2016).

### 3.5 Other tumors

#### 3.5.1 Skin cancer

Expressions of miR-203 are downregulated in skin squamous cell carcinoma. Its upregulation inhibits polycomb repressive complex-1 (PRC1) expressions and blocks the Wnt/β-catenin signaling pathway, thereby suppressing cell proliferation, migration, as well as invasion, and promoting apoptosis (Ting et al., 2020). Circ_0000376 acts as a competitive endogenous RNA to bind miR-203 and regulates miR-203 target gene expressions. TBMS-1 potentially inhibits skin cancer cell proliferations, migration, as well as invasion and accelerates apoptosis by downregulating circ_0000376 and promoting miR-203 expressions (Wang et al., 2021a).

#### 3.5.2 Leukemia

Many somatic cells, specifically in the hematopoietic system, undergo apoptosis. It is a component of terminal differentiation of hematopoietic cells, and inhibition of apoptosis is the major reason for leukemic cell accumulation. TBMS-1 (20 μmol/L) stimulated leukemic K562 cells to exhibit typical morphological characteristics of apoptotic cells, inhibited their proliferation and blocked them in the G2/M phase (Liu et al., 2006). In another study, 15 μmol/L TBMS-1 blocked the cell cycle of leukemic HL60 cells *in vitro*, and its mechanisms may be associated with downregulated Cyc B1 levels (Hu et al., 2003).

Clinically useful cancer chemotherapeutic drugs can induce leukemic cell proliferation *in vitro*. Thus, antileukemic drugs may exert their efficacies via combined synergistic effects of cytotoxicity and induced differentiation. It was previously demonstrated that 2.4 μmol/L TBMS-1 stimulated HL-60 cells to differentiate into more mature cells with functional features of granulocytes. As such, TBMS-1 may be one of these antileukemic drugs (Yu et al., 1996).

#### 3.5.3 Melanoma

Melanoma is an increasingly common and potentially fatal malignant tumor. Single or combined anticancer drugs have been used to prevent its proliferation, metastasis, and spread. TBMS-1 has strong anti-proliferation effects on malignant melanoma cells. TBMS-1 (20 and 40 μmol/L) effectively results in apoptosis of melanoma A375 cells by blocking the cell cycle in the G2/M phase, downregulating Bcl-2*,* and upregulating Bax (Rasul et al., 2012). Another study verified that TBMS-1 inhibits cell proliferations and results in toxic stress to cancer cells by promoting protein-tyrosinephosphatase1B (PTP1B) expressions in melanoma and activating the mitogen-activated protein kinase 1/2- extracellular signal-regulated kinase 1/2 (MEK1/2-ERK1/2) cascade pathway (Du et al., 2020). Small molecular drugs targeting the programmed cell death ligand 1 (PD-L1)/programmed cell death protein 1 (PD-1) axis are effective for enhancing antitumor immunity. TBMS-1 selectively binds mTOR kinase and suppresses mTORC1 activation to dysregulate PD-1/PD-L1 interactions and improve T cell cytotoxicity towards cancer cells by suppressing PD-L1 (Liu et al., 2021).

As discussed above, TBMS-1 can kill multiple types of tumor cells including lung cancer, liver cancer, and cervical cancer. Findings from such studies have provided a foundation for future development of cancer treatments. Studies have shown that TBMS-1 has multiple targets and its effects are mediated by numerous mechanisms such as, inhibition of proliferation, invasion, metastasis, angiogenesis, induction of cell apoptosis, autophagy, and differentiation. Therefore, TBMS-1 may reduce the risk of drug resistance which is commonly seen in single-targeted drugs. Currently, the antitumor effect of TBMS-1 has largely been investigated in *in vitro* settings. Thus, additional *in vivo* experiments are needed to comprehensively and systematically explore the antitumor effects of TBMS-1, and provide adequate scientific reference to promote its clinical application.

Drug resistance is a major obstacle to the treatment of malignant tumors (Hussain et al., 2019). TBMS-1 confers good chemotherapeutic effect and decreases drug resistance via improving the sensitivity of cancer cells to some chemotherapeutic drugs. At low concentrations, TBMS-1 can reverse drug resistance without causing toxic effects (Liu et al., 2011b; Wang et al., 2020b). This suggests that formulations with low concentrations of TBMS-1 should be explored for potential clinical application.

Tumor microenvironment (TME) provides the “soil” needed for the survival of tumor cells. The TME contains cancer-associated fibroblasts (CAFs), extracellular matrix (ECM), immune cells, and related cytokines. Moreover, TME tends to be anoxic and acidic (Turley et al., 2015). Research has now focused on developing cancer drugs targeting the TME. In recent years, several components of the traditional Chinese medicines, such as the main active component of Albiziae Cortex, diosmin, have been widely used in the clinical treatment of tumor (Liu et al., 2016).

TBMS-1 has been reported to confer beneficial effects on the TME. Particularly, TBMS-1 can reduce the expression of MMP, inhibit the degradation of ECM, block endothelial cells from entering the tumor stroma, and inhibit the migration of tumor cells (Yu et al., 2008; Zhong et al., 2016). In addition, TBMS-1 can improve the immune status of TME by inhibiting NF- κ B pathway and reducing the release of inflammatory cytokines such as TNF- *a* (Yin et al., 2011). Targeting PD-L1/PD-1 axis by small-molecule drug is an attractive approach to enhance antitumor immunity. TBMS-1 selectively binds mTOR kinase and suppresses mTORC1 activation to prevent PD-1/PD-L1 interactions and improve T cell cytotoxicity towards cancer cells (Liu et al., 2021). Angiogenesis can cause tumor growth and metastasis by providing nutrition and oxygen to tumor cells. TBMS-1 can downregulate the expression of VEGF through overexpression of miR-126-5p, inhibit angiogenesis in TME, improve microenvironment and inhibit tumor cell proliferation (Shi et al., 2018a; Shi et al., 2018b).

The TBMS-1 inhibits ECM degradation, improves immune cell function, and reduces tumor angiogenesis, thereby preventing tumor development. Several issues relating to the effects of TBMS-1 on the TME, as reported in previous studies, need to be solved. First, the design of most studies is not rigorous and discussion of the mechanism of action is not detailed. Second, most of the previous studies are based on animal models and *in vitro* cell experiments. Third, there are no effective methods to deliver active components to the TME. In future research, studies should explore the mechanism and effect of TBMS-1 on TME, especially on the CAFs and hypoxiainduciblefactor-1 *a* (HIF-1 α) signal pathway. Secondly, nanoparticles/liposomes and light-driven technology should be adopted to improve the therapeutic strategy of TBMS-1 targeting TME. In addition, combinations of traditional Chinese medicine and modern medicine should be formulated to target TME. Thus, cross-disciplinary research should be encouraged to promote effective transformation of laboratory results into clinical practice, improve tumor prevention and treatment.

## 4 Pharmacokinetics

Pharmacokinetic studies are pivotal in drug research and development. It provides systematic concentration and exposure time of the drug as well as predicts the processes associated with its efficacy and toxicity (Xie et al., 2019). Unlike pharmacological studies, pharmacokinetic studies of TBMS-1 have not matured.

TBMS-1 distribution in rats is described as a two-compartment model following intravenous and oral administration. After intravenous administration, elimination half-life (t1/2*β*) of TBMS-1 was 6.70 ± 1.90 h. System clearance rate (CL) was 0.080 ± 0.027 L/h/kg, which was significantly lower than that of rat liver blood flow (3.3 L/h/kg), indicating extremely slow substance clearance by the liver (Davies and Morris, 1993). Volume distribution at the terminal phase (V) was 0.106 ± 0.039 L/kg, which was lower than total moisture (0.67 L/kg), suggesting limited TBMS-1 distribution in the extravascular system. After oral administration, the time to reach the maximum blood concentration was 2.75 ± 0.96 h, while absolute oral bioavailability of TBMS-1 was 0.23 ± 0.13%. This indicates poor absorption of TBMS-1 through the gastrointestinal tract or potential degradation by the acid (Liang et al., 2007). Therefore, intravenous administration may be optimal.

In a previous study, a sensitive and effective technique was developed for measurement of TBMS-1 in rat plasma. The technique integrates qualitative and quantitative benefits of liquid chromatography coupled with hybrid ion trap-time of flight mass spectrometer (HPLC-DAD-IT-TOF-MS) and triple quadrupole-linear ion trap mass spectrometer 5,500 (QTrap 5,500). This approach has been successfully applied in pharmacokinetic studies of oral TBMS-1 in rats. The maximum concentration (C_max_) of TBMS-1 in plasma (1,342 ± 404.50 ng/ml) was achieved at 2.85 ± 1.69 h (T_max_). AUC (0–24 h) and AUC (0-∞) were 8,800.03 ± 2,282.47 and 9,904.41 ± 3,447.84 mg/L*h, respectively. Moreover, MRT_(0–24h)_ was 7.02 ± 1.85 h, and terminal half-life (t_1/2_) was 4.60 ± 3.88 h (Li et al., 2018).

Toxicity or pharmacokinetic curves for a few drugs vary in different species (Tatum-Gibbs et al., 2011; Hu and Smith, 2016). Mice are the most commonly used model species in preclinical efficacy, toxicology, biodistribution, and pharmacokinetic studies. During the early stages of new drug discovery, mice can be used to assess the potential anticancer drugs (Watanabe et al., 2015). Notably, the pharmacokinetics and bioavailability of TBMS-1 in ICR mice were first reported in 2018. They found that t_1/2z_ for oral administration was 2.3 ± 0.5 h, whereas that of intravenous administration was 6.8 ± 5.6 h. After oral administration, T_max_ was 1.8 ± 1.3 h with absolute availability of only 1.0% (Chen et al., 2018). Elsewhere, after intramuscular injection, TBMS-1 levels were highest in the liver and spleen, followed by blood, lung, and heart, and lowest in the kidney and brain. Binding rates for TBMS-1 to plasma, liver, and kidney tissue proteins were 17.1%, 28.1%, and 20.35%, respectively. Therefore, the relative specificity of this distribution implies that TBMS-1 can be used for tumor treatment (Wang et al., 1994).

Pharmacokinetic studies of TBMS-1 are summarized in Table 4. Theoretically, pharmacokinetic parameters for TBMS-1 should include absorption, distribution, metabolism and excretion. However, detailed pharmacokinetic studies on TBMS-1, especially with regards to *in vivo* absorption, the enzymes involved in its metabolism and metabolite chemistry are lacking. Studies should aim at elucidating the pharmacokinetics of TBMS-1 to provide insights on its clinical safety and rational use.

**TABLE 4:** The pharmacokinetics of TBMS-1.

Drugs/Administration	Species	Dose	Detail	References
TBMS-Ⅰ, i.v	Rats	5 mg/kg	t1/2*β* = 6.70 ± 1.90 h; CL = 0.080 ± 0.027 L/(h kg); V = 0.106 ± 0.039 l/kg	Liang et al. (2007)
TBMS-Ⅰ, p.o	Rats	50 mg/kg	T_max_ = 2.75 ± 0.96 h; the absolute oral bioavailability was 0.23 ± 0.13%	Liang et al. (2007)
TBMS-Ⅰ, p.o	Rats	50 mg/kg	C_max_ = 1,342 ± 404.50 ng/ml; Tmax = 2.85 ± 1.69 h; AUC_(0–24h)_ and AUC_(0-∞)_ were 8,800.03 ± 2,282.47, and 9,904.41 ± 3,447.84 mg/L*h, respectively; MRT_(0–24h)_ = 7.02 ± 1.85 h and the terminal half-life (t_1/2_) = 4.60 ± 3.88 h	Li et al. (2018)
TBMS-Ⅰ, i.v	ICR mice	5 mg/kg	t_1/2z_ = 6.8 ± 5.6 h	Chen et al. (2018)
TBMS-Ⅰ, p.o	ICR mice	20 mg/kg	t_1/2z_ = 2.3 ± 0.5 h; T_max_ = 1.8 ± 1.3 h; the absolute availability was 1.0%	Chen et al. (2018)
TBMS-Ⅰ, i.m	Mice	20 mg/kg	Tissue concentration: liver > spleen > blood > lung > heart > kidney > brain; the binding rates of TBMS-Ⅰ with plasma, liver and kidney tissue proteins were 17.1%, 28.1%, and 20.35%, respectively	Wang et al. (1994)

## 5 Toxicity

**FIGURE 3:**
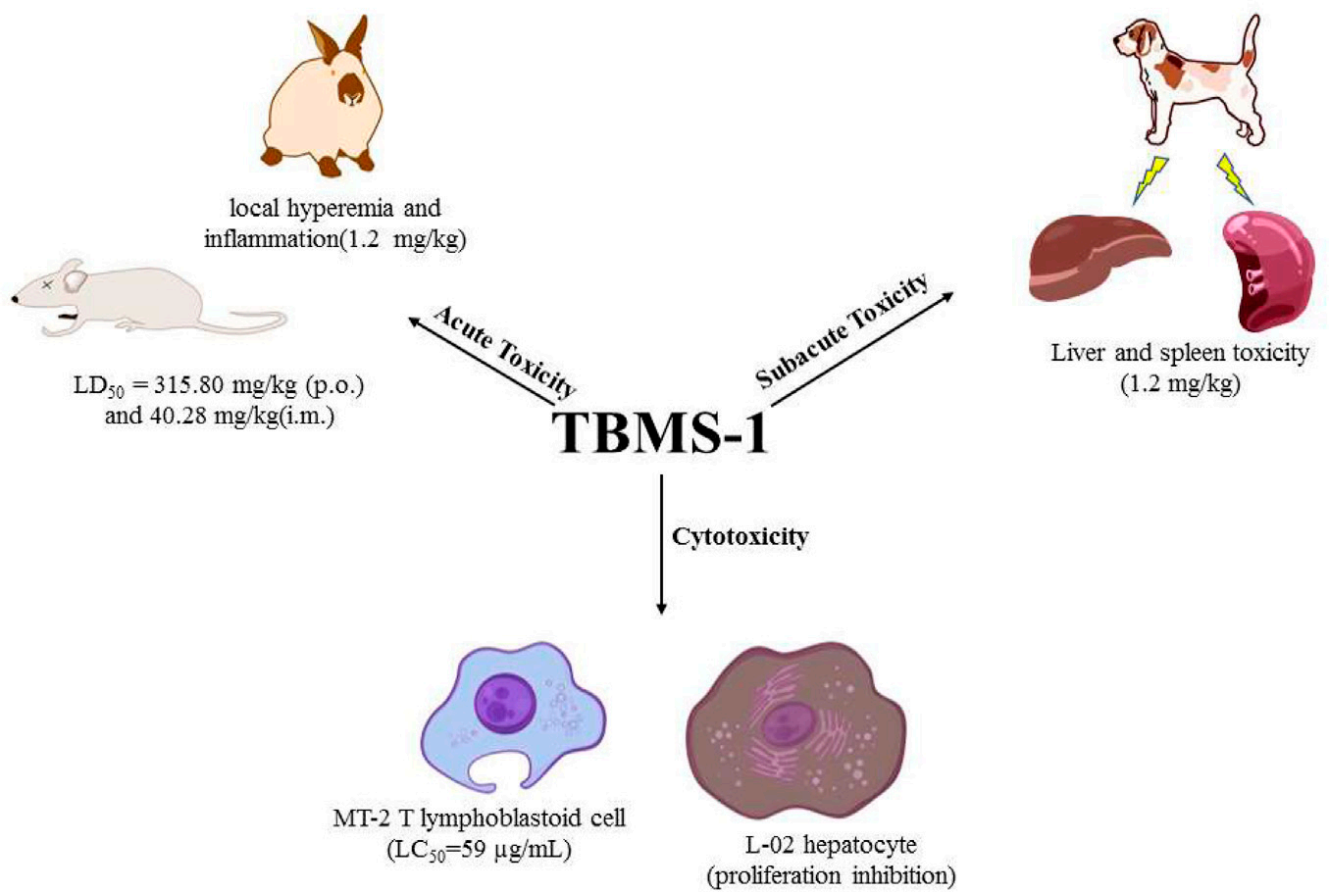
Toxicity of TBMS-1.

When evaluating drug efficacy, their toxicity and safety should also be assessed. In the past few decades, studies have investigated the safety and toxicity profiles of TBMS-1 (Table 5). In general, TBMS-1-associated acute toxicity, sub-acute toxicity, liver and spleen toxicity have attracted a lot of debate with regards to clinical applications of TBMS-1 (Figure 3).

**TABLE 5:** The toxicity of TBMS-1.

	Cell lines/Model	Activity/mechanisms of action	Application	References
Acute toxicity	ICR mice	LD_50_ = 315.80 mg/kg (p.o.) and 40.28 mg/kg (i.m.)	*In vivo*	Yu et al. (1994a)
	Rabbits	0.08 mg/kg (i.m.): no substantial lesion; 1.2 mg/kg (i.m.): local hyperemia and inflammatory stimulation could be produced, and severe cases had brown degeneration	*In vivo*	Fu et al. (1985)
Subacute toxicity Cytotoxicity	Dogs	1.2 mg/kg (i.m.): punctate necrosis of the liver tissue and bleeding of the spleen	*In vivo*	Wang et al. (1984)
	MT-2 cells	LC_50_ = 59 μg/ml	*In vitro*	Yu et al. (1994b)
	L-02 cells	Inhibition of growth of L-02 cells by mitochondrial pathway	*In vitro*	Wang et al. (2011a)

### 5.1 Acute toxicity

Acute toxicity refers to the toxic effects of foreign compounds on the body (human or experimental animals) once (or multiple times within 24 h) after exposure. Related research evaluated the acute toxicity evaluated the acute toxicity of orally and intramuscularly administered TBMS-1 in ICR mice models. The LD_50_ values for TBMS-1 were 315.80 mg/kg and 40.28 mg/kg, respectively (Yu et al., 1994a). In rabbits, a single intramuscular administration of TBMS-1 (0.08 mg/kg) did not cause significant damage. However, a one-time injection of 1.2 mg/kg (TBMS-1) induced local hyperemia and inflammation, suggesting that large doses of TBMS-1 may cause adverse effects (Fu et al., 1985).

### 5.2 Subacute toxicity

Subacute toxicities are toxic manifestations between acute toxicity and chronic toxicity. They are associated with functional or structural damage of the body that occurs after exposure to drugs for a short time period (tens of days or months). The subacute toxicity test was performed in experimental dog models. TBMS-1 was intramuscularly administered once a day at doses of 0.3, 0.6, and 1.2 mg/kg, 5 days a week for 4 weeks. At doses of 0.3 and 0.6 mg/kg, TBMS-1 did not exert any obvious adverse effects on the blood system, liver and kidney functions. After dissection, spleens were established to be enlarged with a purple color. In the high-dose group, spleens of some animals were hemorrhagic with a dark purple color, while liver tissues exhibited punctate necrosis (Wang et al., 1984). These findings are in tandem with those of the pharmacokinetic study, and were attributed to elevated TBMS-1 levels in the liver and spleen.

### 5.3 Cytotoxicity

Toxic effects of TBMS-1 on the T lymphoblastoid cell line (MT-2 cells) have been assessed. At 80 μg/ml to 40 μg/ml, toxic effects of TBMS-1 to MT-2 cells rapidly decreased, and its median lethal concentration (LC_50_) was 59 μg/ml (Yu et al., 1994b). Biologically, TBMS-1 is preferentially distributed in the liver, therefore, if it is systematically administered for cancer treatment, it may damage normal hepatocytes. In a previous study, TBMS-1 dose- and time-dependently inhibited the proliferations of normal hepatocytes (L-02 cells). The mechanisms through which TBMS-1 exerted its inhibitory effects on L-02 cells were associated with the mitochondrial pathway. The inherent mechanisms are collapse of mitochondrial membrane potential, release of Cyt-c from the mitochondria to the cytoplasm, activation of Caspase-9 and Caspase-3, suppression of Bcl-2 and elevation of Bax (Wang et al., 2011a).

Although TBMS-1 has good antitumor effects, its toxic effects should be evaluated to in clinical applications. The toxicological effects of TBMS are classified as acute toxicity, subacute toxicity, and cytotoxicity. Acute and subacute toxicity studies have shown that TBMS-1 is less toxic at low doses as opposed to high doses. This implies that the potential toxicity of TBMS-1 on the liver and spleen should be assessed during clinical applications.

The mechanisms underlying the cytotoxic and adverse effects of TBMS-1 are similar to those driving its antitumor activities. Some anticarcinogens meant to kill cancer cells may also cause damage to normal cells. Moreover, natural chemicals derived from plants are thought to be less toxic to normal cells than chemosynthetic drugs (Li et al., 2015; Gezici and Sekeroglu, 2019).

## 6 Targeting preparation

Pharmacological studies have shown that TBMS-1 exert anti-tumor effects. However, it causes toxic and irritation effects which have limited its clinical adoption. Currently, several anticancer drugs, antiviral drugs, and genetic drugs have been used to prepare targeted drug delivery systems (Zhang et al., 2022; Zhu et al., 2022). Such systems have high specificity and selectivity, can reduce drug dosage and administration times, as well as effectively alleviate toxicity and improve drug efficacy. Therefore, targeting preparations should be developed to improve efficacy of TBMS-1 while reducing its toxic effects. In this review, we discuss liver- and lung-targeting preparations of TBMS-1 which have been extensively studied.

### 6.1 Liver-targeting preparations

Poly (n-butyl cyanoacrylate)-TBMS-1 nanoparticles were successfully prepared and their therapeutic effects were compared to those of TBMS-1. Inhibitory rates of the prepared nanoparticles and TBMS-1 on H_22_ hepatocellular carcinoma in mice were comparable. Irritation effects of TBMS-1 nanoparticles on blood vessels were markedly decreased, while its LD_50_ increased by about 20% (Li et al., 2004). In another study, TBMS-1 nanoparticles were found to be mainly distributed in the liver, however, their toxic effects to the lungs and liver were significantly low, relative to those of TBMS injections. Its LD_50_ value was 13.5% high, while its vascular irritation effects were much lower, relative to those of TBMS injections (Sun et al., 2005).

### 6.2 Lung-targeting preparations

Lung cancer cells are highly sensitive to TBMS-1. Preparation of lung-targeting microspheres based on TBMS-1 characteristics can reduce the effective dose, thereby decrease the toxic and side effects of the microspheres to other tissues and enhance its efficacy.

The results of tumor inhibition experiment showed that Lewis lung cancer cells were highly sensitive to TBMS-1. In the animal experiment of liver targeting nanoparticles of TBMS-1, it was found that even if the liver targeting preparation was made, the concentration of the drug in the lungs was very large (Li et al., 2004). Therefore, it is important to experimentally study lung-targeting microspheres of TBMS-1. In addition, pharmacological comparative studies should be conducted to and targeting evaluation between it and the original drug.

In a previous study, lung-targeting microspheres of TBMS-1 were prepared via the double emulsion method. The encapsulation efficiency of TBMS-1 was determined by high performance liquid chromatography (HPLC), after which the optimized preparation conditions were investigated through single factor experiments. Finally, TBMS-1 lung-targeting microspheres with the required particle sizes were obtained (Li et al., 2005). The *in vitro* release of TBMS-1 lung-targeting microspheres were evaluated by the dynamic dialysis technique. *In vitro* release of the simple mixture of TBMS-1 and carrier material (polylactic-glycolic acid copolymer) conformed to the first-order kinetic equation. Besides, *in vitro* release of drug-loaded lung-targeting microspheres formed by TBMS-1-polylactic-glycolic acid copolymer were in accordance with the Weibull equation, and the half-life was significantly prolonged with a sustained release effect (Li et al., 2009).

The development of targeting preparations for TBMS-1 provides an effective approach for liver and lung cancer treatment, which also provides good ideas and methods for treatment of other cancers. Second, in addition to nanoparticles and microspheres, more dose forms can be designed to increase the variety of targeting preparations, so as to adapt to treatment of different organs and tissues.

## 7 Discussion and conclusion

The serious adverse effects of traditional anticarcinogens and drug resistance have necessitated the development of more effective anticarcinogens (Katz and Shaked 2015; Bar-Zeev et al., 2017). Understanding the mechanisms driving tumor development is a prerequisite to identifying the mechanisms of action for traditional therapeutic approaches and development of new drugs (Efferth et al., 2007). TBMS-1 has been shown to exert antitumor effects in multiple cancers. Therefore, TBMS-1 is a promising natural substance for cancer treatment. Majority of studies on TBMS-1 have been conducted using *in vitro* experiments. Hence, future *in vivo* studies are needed to further explore the antitumor effects and mechanisms of TBMS-1.

P38 is a member of the MAPK signaling pathways with diverse effects in different cancers. Moreover, its effects vary depending on the tumor stage (Maik-Rachline et al., 2020). The p38 MAPK sub-family has four main isoforms, each with different selectivity and specificity for substrates. For this reason, the tissue distribution of p38 in different tumor cells vary (Yokota and Wang 2016). TBMS-1 induces apoptosis of ovarian cancer, choriocarcinoma, and prostate carcinoma cells by activating the p38 pathway. However, it has not been determined whether TBMS-1 inhibits the proliferation of other cancer cells via this pathway. Further studies should explore the antitumor mechanisms of TBMS-1 in different cancer cells y using highly specific antibodies of p38 isoform.

Cholesterol plays an important role in saponin-induced cytotoxicity. Saponins aggregate with membrane cholesterol to cause changes in cell membrane permeability, destruction of the lipid raft, and damage to the mitochondrial membrane. The also activate programmed cell death-related signaling pathways (Lorent et al., 2014). Tumor cells are highly sensitive to saponins-induced cytotoxicity because they contain higher levels of intracellular cholesterol and lipid rafts compared with normal cells (Shaheen et al., 2018; Mollinedo and Gajate 2020). The selective cytotoxicity of TBMS-1 for cancer cells makes it relatively safe as an anticancer agent. However, at high doses, TBMS-1 has been reported to induce toxic effects.

Pharmacokinetics refers to the quantitative estimation of absorption, distribution, metabolism, and excretion properties of drugs within a living organism. We found that TBMS-1 was widely distributed in many tissues *in vivo*, and this may explain its high anticancer effects. After oral administration, TBMS-1 is easily degraded in the gastrointestinal tract, resulting in decreased bioavailability. Therefore, intravenous or intramuscular administration should be considered in clinical applications. To date, the enzymes responsible for TBMS-1 metabolism (oxidation, and dehydrogenation among others) and the resultant metabolites have not been elucidated. Therefore, additional pharmacokinetic studies are advocated to clarify this. Meanwhile, studies have shown that TBMS-1 is converted into metabolites under certain conditions *in vivo*. In addition, the pharmacodynamic characteristics of TBMS-1 have not been revealed. This calls for investigations to determine the pharmacodynamic profile of TBMS-1 and evaluate the antitumor activities and toxicity its metabolites.

It is necessary to develop suitable approaches for reducing the toxic effects of TBMS-1 to improve its medicinal value. An important approach is structural modification of TBMS-1 to create derivatives with high efficacy and low toxicity. Another approach of reducing toxic effects involves the formulation of drug combinations. When combined with other anticancer drugs, the anticancer effects of TBMS-1 are potentiated. For instance, a combined use of TBMS-1 and cisplatin improved the sensitivity of cancer cells to cisplatin, and reduced the dose, improved efficacy and reduced toxicity. In addition, targeting preparations have been shown to reduce the TBMS-1-associated toxic effects. Currently, nanoparticle liver-targeting preparations and lung-targeting microsphere preparations have been shown to enhance the specificity of TBMS-1 to target organs, reduce its wide distribution, toxic effects, and maintain drug concentration at the target organs. Moreover, such preparations provide sustained release effect. In view of this, development of targeting preparations of TBMS-1 is expected to be an important focus in future research.
